# Supplementary material for: Neutrophil-to-Lymphocyte Ratio and Cytokine Profiling as Predictors of Disease Severity and Survival in Unvaccinated COVID-19 Patients
Source: Vaccines (Basel). 2024 Jul 31;12(8):861. doi: 10.3390/vaccines12080861 (PMC11360520; doi:10.3390/vaccines12080861)
Supplement: Supplementary file 1 [file vaccines-12-00861-s001.zip › vaccines-3079941-supplementary.pdf]

vaccines

MDPI

Table S1. Prevalence of Various Comorbidities in Non-Intubated and Intubated Patients (Expressed as Percentages).

Comorbidity	Non-Intubated (%)	Intubated (%)
Hypertension	35.8	38.5
Nephropathy	3.1	7.5
Diabetes mellitus	22.8	29.8
Cerebrovascular event	0.6	0.0
Epilepsy	0.6	0.0
Cardiopathy	9.9	10.1
Obesity	28.4	32.3
Overweight	24.7	33.5
Hepatopathy	3.1	3.1
Alcoholism	0.6	3.1
Chronic Bronchitis	0	1.9
Obstructive sleep apnea syndrome	0.6	0.6
Asthma	0.6	1.9
Oncological	5.6	2.5
Chronic obstructive pulmonary disease	3.1	5.0
Smoking	10.5	11.2
Thyroid Disease	7.4	3.1

This table shows the prevalence of various comorbidities in intubated and non-intubated patients, expressed as percentages. The data suggest a similar distribution of comorbidities between the two groups, indicating that preexisting conditions do not significantly influence the decision to intubate within the context of this study.

Table S2. Prevalence of Various Comorbidities in Non-Surviving and Surviving Patients (Expressed as Percentages).

Comorbidity	Non-Surviving (%)	Surviving (%)
Hypertension	35.9	37.7
Nephropathy	8.7	3.6
Diabetes mellitus	35.9	21.8
Cerebrovascular event	0.0	0.5
Epilepsy	0.0	0.5
Cardiopathy	9.7	10.0
Obesity	23.3	33.6
Overweight	37.9	25.0
Hepatopathy	1.9	3.6
Alcoholism	2.9	1.4
Chronic Bronchitis	1.0	0.9
Obstructive sleep apnea syndrome	0.9	0.5
Asthma	1.0	0.9
Oncological	2.9	4.5
Chronic obstructive pulmonary disease	5.8	3.2
Smoking	10.7	10.9
Thyroid Disease	5.8	5.0

This table shows the prevalence of various comorbidities in surviving and deceased patients, expressed as percentages. The data suggest a similar distribution of comorbidities between the two groups, indicating that preexisting conditions do not significantly influence the survival outcomes within the context of this study.
